# Supplementary material for: Hughes Abdominal Repair Trial (HART)—abdominal wall closure techniques to reduce the incidence of incisional hernias: feasibility trial for a multicentre, pragmatic, randomised controlled trial
Source: BMJ Open. 2017 Dec 19;7(12):e017235. doi: 10.1136/bmjopen-2017-017235 (PMC5778308; doi:10.1136/bmjopen-2017-017235)
Supplement: Author's manuscript [file bmjopen-2017-017235.draft_revisions.pdf]

BMJ Open

BMJ Open is committed to open peer review. As part of this commitment we make the peer review history of every article we publish publicly available.

When an article is published we post the peer reviewers' comments and the authors' responses online. We also post the versions of the paper that were used during peer review. These are the versions that the peer review comments apply to.

The versions of the paper that follow are the versions that were submitted during the peer review process. They are not the versions of record or the final published versions. They should not be cited or distributed as the published version of this manuscript.

BMJ Open is an open access journal and the full, final, typeset and author-corrected version of record of the manuscript is available on our site with no access controls, subscription charges or pay-per-view fees (http://bmjopen.bmj.com).

If you have any questions on BMJ Open's open peer review process please email editorial.bmjopen@bmj.com

BMJ Open

Hughes Abdominal Repair Trial (HART) - Abdominal wall closure techniques to reduce the incidence of incisional hernias: Feasibility study for a multi-centre pragmatic randomised controlled trial

Journal:	*BMJ Open*
Manuscript ID	bmjopen-2017-017235
Article Type:	Research
Date Submitted by the Author:	11-Apr-2017
Complete List of Authors:	Harries, Rhiannon; University Hospital of Wales, Department of Colorectal Surgery; Welsh Barbers Research Group<br>Cornish, Julie; Welsh Barbers Research Group; Royal Glamorgan Hospital, Department of Colorectal Surgery<br>Bosanquet, David; University Hospital of Wales, Department of Colorectal Surgery; Welsh Barbers Research Group<br>Rees, Buddug; University Hospital of Wales, Department of Colorectal Surgery<br>Ansell, James; University Hospital of Wales, Department of Colorectal Surgery; Welsh Barbers Research Group<br>Frewern, Natasha; University Hospital of Wales, Department of Colorectal Surgery; Welsh Barbers Research Group<br>Dhruva Rao, Pawan; University Hospital of Wales, Department of Colorectal Surgery; Welsh Barbers Research Group<br>Phillips, Simon; University Hospital of Wales, Department of Colorectal Surgery<br>Morris, Chris; University Hospital of Wales, Department of Colorectal Surgery<br>Horwood, James; University Hospital of Wales, Department of Colorectal Surgery<br>Davies, Mansel Leigh; University Hospital of Wales, Department of Colorectal Surgery<br>Davies, Michael ; University Hospital of Wales, Department of Colorectal Surgery<br>Hargest, Rachel; University Hospital of Wales, Department of Colorectal Surgery<br>Islam, Saiful; Swansea University, Trials Unit<br>Bashir, Nadim; Swansea University, Trials Unit<br>Watkins, Alan; Swansea University, Trials Unit<br>Jones, SIan; Involving People, Health and Care Research Wales<br>Russell, Ian; Swansea University, Trials Unit<br>Torkington, Jared; University Hospital of Wales, Department of Colorectal Surgery
**Primary Subject Heading**:	Surgery
Secondary Subject Heading:	Evidence based practice

For peer review only - http://bmjopen.bmj.com/site/about/guidelines.xhtml

Keywords:

For peer review only

Hughes Abdominal Repair Trial (HART) - Abdominal wall closure techniques to reduce the incidence of incisional hernias: Feasibility study for a multi-centre pragmatic randomised controlled trial (ISRCTN 25616490)

Harries RL[1,2], Cornish J[2,3], Bosanquet D[1,2], Rees B[1], Ansell J[1,2], Frewer N[1,2], Dhruva Rao P[1,2], Phillips SM[1], Morris C[1], Horwood J[1], Davies ML[1], Davies MM[1], Hargest R[1], Islam S[4], Bashir N[4], Watkins A[4], Jones S[5], Russell IT[4], Torkington J[1] ; on behalf of the HART Trial Management Group

1 University Hospital of Wales, Cardiff

2 Welsh Barbers Research Group

3 Royal Glamorgan Hospital, Llantrisant

4 Swansea Trials Unit, Swansea University

5 Involving People, Health and Care Research Wales

Correspondence should be addressed to:

Prof Jared Torkington, Dep. of Surgery, University Hospital of Wales, Heath Park, Cardiff CF14 4XW

jared.torkington@wales.nhs.uk Tel: +44 2920 747747

For peer review only

Word count: 1892

Article type: Randomised controlled trial

Running title: Hughes Abdominal Repair Trial: Feasibility study

Conflict of interest: None declared

Keywords: Incisional hernia; Abdominal closure; Hughes repair; Mass closure; randomised controlled trial; colorectal cancer

Abstract

Objectives: Incisional hernias are common complications of midline abdominal closure. The 'Hughes Repair' combines a standard mass closure with a series of horizontal and two vertical mattress sutures within a single suture. There is evidence to suggest this technique is as effective as mesh repair for the operative management of incisional hernias; however, no trials have compared Hughes Repair with standard mass closure for the prevention of incisional hernia formation. This paper aims to test the feasibility of running a randomised controlled trial of a comparison of abdominal wall closure methods following midline incisional surgery for colorectal cancer.

Design and Setting: A feasibility study (with 1:1 randomisation) conducted perioperatively during colorectal cancer surgery.

Participants: Patients undergoing midline incisional surgery for resection of colorectal cancer.

Interventions: Comparison of two suture techniques (Hughes repair or standard mass closure) for the closure of the midline abdominal wound following surgery for colorectal cancer.

Primary and Secondary Outcomes: A 30-patient feasibility study assessed recruitment, randomisation, deliverability and early safety of the surgical techniques used.

Results: A total of 30 patients were randomised from 43 patients recruited and consented, over a 5-month period. 14 and 16 patients were randomised to Arm A and B, respectively. There was 1 superficial surgical site infection (SSI) and 2 organ space SSI reported in arm A and 2 superficial SSI and 1 complete wound dehiscence in arm B. There were no suspected unexpected serious adverse reactions reported in either arm. Independent data monitoring committee found no early safety concerns.

Conclusions: The feasibility study found no early safety concerns and demonstrated that the trial was acceptable to patients. Progression to the pilot and main phases of the trial has now commenced following approval by the independent data monitoring committee.

Trial registration: ISRCTN 25616490

For peer review only

Strengths and Limitations of this study

- The results of this feasibility study demonstrate that recruitment to a randomised controlled trial comparing suturing techniques in midline incisions following colorectal cancer resection surgery is acceptable to patients with no early safety concerns identified
- This feasibility study is not powered for a definitive study and simply reports the recruitment, deliverability and safety
- We report blinded outcome data and have not included incisional hernia rates in order to prevent the introduction of bias or reduction of equipoise for future recruitment of the main trial

For peer review only

Introduction

Incisional hernias are common complications of midline abdominal incisions, with a reported incidence of 12.8% at 2 years follow-up [1]. They can result in significant morbidity, impaired quality of life [2] and frequently require emergency surgery. Despite recent development in mesh technology, incisional hernia repair still has disappointingly high recurrence rates (up to 54% in suture repair and up to 36% in mesh repair [3-4]). Prevention of the development of incisional hernia therefore brings significant benefits for both patients and healthcare provision funding.

'Mass closure' remains the standard technique for abdominal closure (closing all layers of the abdominal wall, excluding the skin), with either non-absorbable or slow-resorbing sutures, such as polydioxanone (PDS) [5]. A systematic review and meta-regression of over 14,000 patients found no difference in incisional hernia rate comparing suture material [1]. This poses the question as to whether improved suture technique may reduce incisional hernia formation. The STITCH trial [6], a Dutch multicentre, randomised controlled trial compared small stitch continuous sutures with large stitch standard mass closure in 560 patients. Results demonstrated a reduction in the rate of incisional hernia from 21% in the large bite group to 13% in the small bite group at one-year follow-up. The CONTINT trial, currently still in recruitment, is comparing continuous with interrupted sutures in closing midline incisions after emergency laparotomy [7].

The eponymously titled 'Hughes Repair' (Professor Les Hughes, 1932-2011 [8]), also known as the 'far-and-near' or 'Cardiff Repair' [9] combines a standard mass closure (two loop 1 PDS sutures) with a series of horizontal and two vertical mattress sutures within a single suture (1 Nylon); theoretically distributing the load along the incision length as well as across it (**Figure 1**). The principles are:

1. To ensure, by palpation, that only sound normal tissues are used for the repair
2. To use graduated tension for easy approximation
3. Use a monofilament Nylon suture, which has the advantage of slipping easily through tissues to create a pulley system [10].

For peer review only

The Hughes Repair has been shown to have outcomes as effective as the standard mesh repair in incisional hernia repairs [11]. It is also used for closing abdomens when patients are at high risk of incisional hernias, after complete abdominal wound dehiscence and laparostomy [12].

This feasibility study aimed to establish whether a randomised controlled trial to compare Hughes Repair with standard mass closure for prevention of midline incisional hernia, in patients undergoing colorectal cancer resectional surgery, would be deemed acceptable to patients, achieve adequate recruitment and result in no early safety concerns.

For peer review only

Methods/Design

Study Design

The HART trial hypothesis is that the Hughes Repair will reduce the incidence of clinically detected incisional hernia at one year in patients undergoing midline abdominal wall closure incisions following elective or emergency colorectal cancer surgery when compared with standard mass closure **Figure 2**. This is a 1:1 randomised controlled trial comparing two suture techniques for the closure of the midline abdominal wound following surgery for colorectal cancer. The study was approved by the Wales Research Ethics Committee (MREC 12/WA/0374).

Aims and outcome measures

The feasibility study aimed to assess the ability of the trial to recruit and consent patients over a 5-month period and the deliverability and safety of the Hughes Repair. Operation specific adverse events collected included surgical site infection and full wound dehiscence. Early surgical safety was assessed using post-operative complications, serious adverse event reporting and wound diaries. In this paper we report blinded outcome data and have not included incisional hernia rates in order to prevent the introduction of bias or reduction of equipoise for future recruitment of the main trial.

Independent data monitoring committee reviewed the un-blinded safety data after completion of the feasibility phase. The outcome measures for the full trial have been previously reported [13]; Primary outcome measure being the rate of incisional hernia at 1-year follow-up assessed by clinical examination.

Eligibility Criteria

Eligibility criteria were assessed at two time points; at initial screening and at point of surgical closure /randomisation. Adult patients (aged 18 year or over), able to give informed consent, undergoing either elective colorectal cancer surgery following full staging investigations including an abdominal CT scan or emergency surgery in those with a strong suspicion of colorectal cancer on abdominal CT scan were eligible at point of initial screening. All patients had to be suitable for either Hughes repair or standard mass closure. At point of surgical

For peer review only

closure, eligibility was further assessed, and all patients who had a midline incision (open or laparoscopic assisted/ converted) of 5cm or more in length were deemed suitable for randomisation. Patients requiring mesh insertion or having an abdominal musculofascial flap for closure of the perineal defect in abdomino-perineal wound closure were excluded.

Consent

Patients were identified, approached and provided with a patient information leaflet. Consent for trial participation, was gained by either consultant surgeons or surgical registrars who had current 'Good Clinical Practice' certification.

Randomisation and Data Collection

An adaptive randomisation design was used to allocate eligible patients to groups of similar size [14]. Patients were randomised in a 1:1 ratio to either Mass closure or Hughes Repair. Randomisation took place during surgery and as close as possible to the time when the surgeon commenced closure. The patient was blinded to the treatment allocation assigned to them. Data management was supported by the Swansea Trials Unit.

Surgical Quality Assurance

To assure the quality of the repair techniques, all surgeons participating in the trial (consultants and registrars) completed training and quality assessment on the Hughes Repair. All participating surgeons were assessed by the Chief Investigator and were approved only when closure technique was satisfactory. A reference instructional video was provided to participating surgeons. To monitor the training of professionals contributing to HART, a log was maintained with details of training, both surgical and in research governance notably 'Good Clinical Practice'. For the purposes of the study, mass closure was taken to be the responsible consultant surgeon's standard closure technique.

Radiological Evaluation of incisional hernia

A dedicated trial radiologist determined whether there was a hernia present on the 1-year colorectal cancer surveillance CT scan. They defined an incisional hernia as herniation of the bowel or other intra-abdominal content outside the abdominal wall, and also identified the

For peer review only

presence of other hernias and the quality of the recti muscle. All scans were performed using the standard departmental protocol for follow-up scans.

Sample size

The feasibility study aimed to recruit a total of 30 patients over a 5-month period. The sample size for the main study has been published previously [13].

Funding

The NIHR Health Technology Assessment programme requested this feasibility study as a prerequisite for awarding grant 12/35/29 for the pilot and main phases of HART.

For peer review only

Results

Recruitment and randomisation

A total of 62 patients were screened and assessed for eligibility for entry into the trial over a 5-month time period (**Figure 3**). Of those screened, 43 patients consented to entry into the trial (69%). A total of 30 of the 43 patients were randomised in the operating theatre (14 patients were randomised to arm A and 16 patients were randomised to arm B). The reasons for exclusion are described in the CONSORT diagram (**Figure 3**).

For the 30 patients who were randomised, the median age was 74 years (IQR 66-78). There were 23 males and 7 females. Demographical data is presented in **Table 1**. In arm A, one patient had died prior to 12-month follow-up and a further patient had transferred care to another unit. In arm B, one patient had died prior to 12-month follow-up.

Safety data

There were a total of 16 serious adverse events reported in 10 patients (**Table 2**); serious adverse event rate was 33.3% in arm A and 31.25% in arm B. There were no suspected unexpected serious adverse reactions reported in either arm. With regard to wound related complications, there was 1 superficial surgical site infection and 2 organ space surgical site infection reported in arm A and 2 superficial surgical site infections and 1 complete wound dehiscence requiring a return to theatre in arm B (**Table 3**). The Data Monitoring Committee (DMC) reviewed the un-blinded adverse events data and identified no safety concerns.

For peer review only

Discussion

The results of this feasibility study demonstrated that a randomised controlled trial designed to compare two suture techniques for the prevention of midline incisional hernia, in patients undergoing cancer resectional surgery, was able to recruit 30 patients over 5 months, as planned. This suggests that the proposed sample size of 800 patients for the main full trial is achievable in the time scale with the proposed number of sites recruiting (approximately 20) [13].

The feasibility study results established that the trial was acceptable to patients. Patient participation rates were high, demonstrated by 69% of all eligible patients consenting to participation in the trial. Nine patients were screened for eligibility but not consented due to staff shortages highlighting the importance of having adequate number of approved consenting staff on the delegation log. Due to the nature of the study it was accepted that not all patients consented would eventually be randomised. In fact, there was a higher than expected number of patients consented and not eventually randomised (31%). Patients were consented but not randomised if the intraoperative procedure performed did not meet the inclusion criteria, for example not midline incision, conversion to open procedure using a non-midline incision, or emergency patients found not to have a tumour intra-operatively.

The serious adverse event rate and wound related complications were similar between both arms and reassuringly there were no suspected unexpected serious adverse reactions reported. It is anticipated that reporting on the full trial will take place in 2019.

For peer review only

List of abbreviations

AAA: Abdominal aortic aneurysm

BMI: Body Mass Index

CONTINT: CONTinuous versus INTerrupted abdominal wall closure after emergency midline laparotomy

COPD: Chronic Obstructive Pulmonary Disease

CT: Computed tomography

DMC: Data Monitoring Committee

HART: Hughes Abdominal Repair Trial

IQR: interquartile range

NIHR: National Institute of Health Research

PDS: polydioxanone

SAEs: Serious Adverse Events

SSI: Surgical Site Infection

STITCH: Suture Techniques to reduce the Incidence of The inCisional Hernia (RCT)

Competing Interests

There are no competing interests to declare

Authors Contributions

RLH, JC, DB, BR, JA, NF, ITR, JT conceived, designed and drafted the initial protocol

SMP, CM, JH, MLD, MMD, RH, SJ, PDR, aided in redrafting and revising the protocol and contributed heavily to completion of the feasibility trial and pilot stages

SI, AW, NB, ITR have written and designed the analysis plan and revised the protocol

JC, DB, ITR and JT led the team that acquired the funding

All authors agree to be accountable for all aspects of the work. All authors read and approved the final manuscript.

For peer review only

References

1. Bosanquet D, Ansell J, Abdelrahman T, Cornish J, Harries R, Stimpson A, Davies L, Glasbey J, Frewer K, Frewer N, Russell D, Russell I, Torkington J. Systematic Review and Meta-Regression of factors affecting midline incisional hernia rates: Analysis of 14,618 patients. PLoS ONE 2015;10(9): e0138745.

2. van Ramshorst GH, Eker HH, Hop WC, Jeekel J, Lange JF. Impact of incisional hernia on health-related quality of life and body image: a prospective cohort study. Am J Surg, 2012;204(2):144-50.

3. Diener MK, Voss S, Jensen K, Büchler MW, Seiler CM. Elective midline laparotomy closure: the INLINE systematic review and meta-analysis. Ann Surg, 2010; 251(5):843-56.

4. van 't Riet M, Steyerberg EW, Nellensteyn J, Bonjer HJ, Jeekel J. Meta-analysis of techniques for closure of midline abdominal incisions. Br J Surg, 2002;89(11):1350-6.

5. Sajid MS, Parampalli U, Baig MK, McFall MR. A systematic review on the effectiveness of slowly-absorbable versus non-absorbable sutures for abdominal fascial closure following laparotomy. Int J Surg, 2011; 9(8):615-25.

6. Deerenberg EB, Harlaar JJ, Steyerberg EW, Lont HE, van Doorn HC, Heisterkamp J, Wijnhoven BP, Schouten WR, Cense HA, Stockmann HB, Berends FJ, Dijkhuizen FP, Dwarkasing RS, Jairam AP, van Ramshorst GH, Kleinrensink GJ, Jeekel J, Lange JF. Small bites versus large bites for closure of abdominal midline incisions (STITCH): a double-blind, multicentre, randomised controlled trial. Lancet. 2015, 386(10000):1254-60.

7. Rahbari NN, Knebel P, Kieser M, Bruckner T, Bartsch DK, Friess H, Mihaljevic AL, Stern J, Diener MK, Voss S, Rossion I, Büchler MW, Seiler CM. Design and current status of CONTINT: continuous versus interrupted abdominal wall closure after emergency midline laparotomy - a randomized controlled multicenter trial [NCT00544583]. Trials, 2012;13:72.

8. Hughes BR & Webster D. Leslie Ernest Hughes OBITUARY. BMJ, 2011. 343.

9. Shukla VK, Gupta A, Singh H, Pandey M, Gautam A. Cardiff repair of incisional hernia: a university hospital experience. Eur J Surg, 1998;164(4):271-4.

For peer review only - http://bmjopen.bmj.com/site/about/guidelines.xhtml

10. Mudge M, Harding KG, Hughes LE. Incisional hernia. Br J Surg, 1986;73(1): 82.

11. Godara R, Garg P, Shankar G. Comparative evaluation of Cardiff repair and mesh plasty in incisional hernias. Internet J Surg [Internet]. 2007; 9 [about 8 p.].

12. Malik R & Scott NA. Double near and far prolene suture closure: a technique for abdominal wall closure after laparostomy. Br J Surg, 2001;88(1):146-7.

13. Cornish J, Harries RL, Bosanquet D, Rees B, Ansell J, Frewer N, Dhruva Rao P, Parry C, Ellis-Owen R, Phillips SM, Morris C, Horwood J, Davies ML, Davies MM, Hargest R, Hilton J, Harris D, Ben-Sassi A, Rajagopal R, Hanratty D, Islam S, Watkins A, Bashir N, Jones S, Russell IR, Torkington J, on behalf of the HART Trial Management Group. Hughes Abdominal Repair Trial (HART) - Abdominal wall closure techniques to reduce the incidence of incisional hernias: Study protocol for a multi-centre pragmatic randomised controlled trial (ISRCTN 25616490). Trials 2016; 17(1):454.

14. Russell D, Hoare ZSJ, Whitaker Rh, Whitaker CJ, Russell IT. Generalised method for adaptive randomisation in clinical trials. Statistics in Medicine 2011; 30: 922-34.

For peer review only

Table 1. Patient demographics and clinical characteristics

	Arm A (N=14)	Arm B (N=16)	Total (N=30)
Gender			
Male	10 (71.4%)	13 (81.3%)	23 (76.7%)
Female	4 (28.6%)	3 (18.7%)	7 (23.3%)
Median Age (IQR)	75 (61-78)	73 (68- 77)	74 (66-78)
Mean BMI (Min-Max)	30 (22-49)	29 (18-42)	29 (18-49)
Smoker	1 (7.1%)	3 (18.8%)	4 (13.3%)
Steroid/ immunosuppression use	0 (0%)	0 (0%)	0 (0%)
Diabetes	5 (35.7%)	4 (25%)	9 (30%)
Connective tissue disorder	1 (7.1%)	0 (0%)	1 (3.3%)
COPD	1 (7.1%)	2 (12.5%)	3 (9.9%)
AAA (known or previous repair)	0 (0%)	0 (0%)	0 (0%)
Previous abdominal surgery	8 (57.1%)	8 (50%)	15 (50%)
Neoadjuvant chemotherapy	1 (7.1%)	0 (0%)	1 (3.3%)
Neoadjuvant radiotherapy	1 (7.1%)	1 (6.3%)	2 (6.6%)
Incisional Hernia present pre-operatively	0 (0%)	1 (6.3%)	1 (3.3%)
Previous incisional hernia repair	1 (7.1%)	0 (0%)	1 (3.3%)
Non incisional hernia present pre-operatively	0 (0%)	3 (18.8%)	3 (9.9%)
Mode of surgery			
Laparoscopic	4 (28.6%)	6 (37.5%)	10 (33.3%)
Laparoscopic converted	7 (50%)	3 (18.8%)	10 (33.3%)
Open	3 (21.4%)	7 (43.7%)	10 (33.3%)

For peer review only

Table 2. Reported serious adverse events

	**Arm A**	**Arm B**
Myocardial infarction	2	2
Lower respiratory tract infection	2	1
Pulmonary embolism	1	0
Renal failure	0	1
Anastomotic leak	2	0
Parastomal hernia	0	1
Superficial surgical site infection	2	0
Dehiscence	0	1
Death*	1	0
**Total SAEs**	**10**	**6**
**Total Patients affected**	**5**	**5**

* 1 SAE reported was reported as ‘death’, therefore it has had to be listed as an event of death. There were two other SAEs that resulted in death within the feasibility study

For peer review only

Table 3. Wound related complications

	**Arm A**	**Arm B**
Superficial SSI	1	2
Deep SSI	0	0
Organ space SSI	2	0
Wound dehiscence	0	1
**Total wound related complications**	**3**	**3**

For peer review only

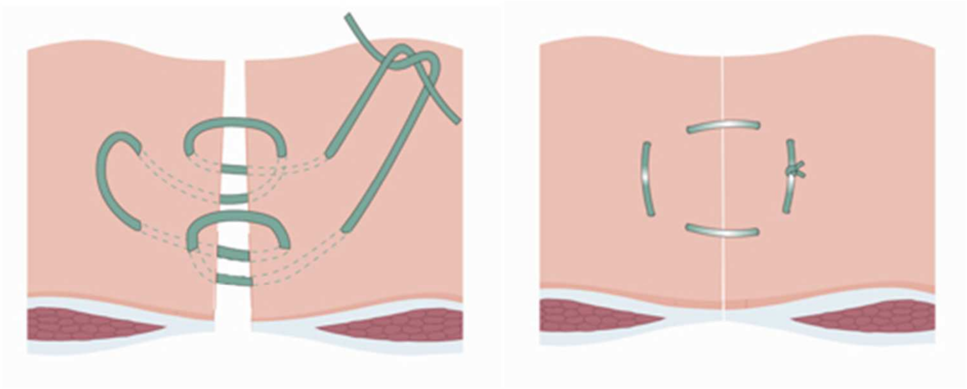

Figure 1. Diagram showing the Hughes closure method, using a combination of standard mass closure with a series of horizontal and two vertical mattress sutures within a single suture. When the sutures are pulled to close the defect, the sutures lie both across and along the incision.

191x79mm (72 x 72 DPI)

For peer review only

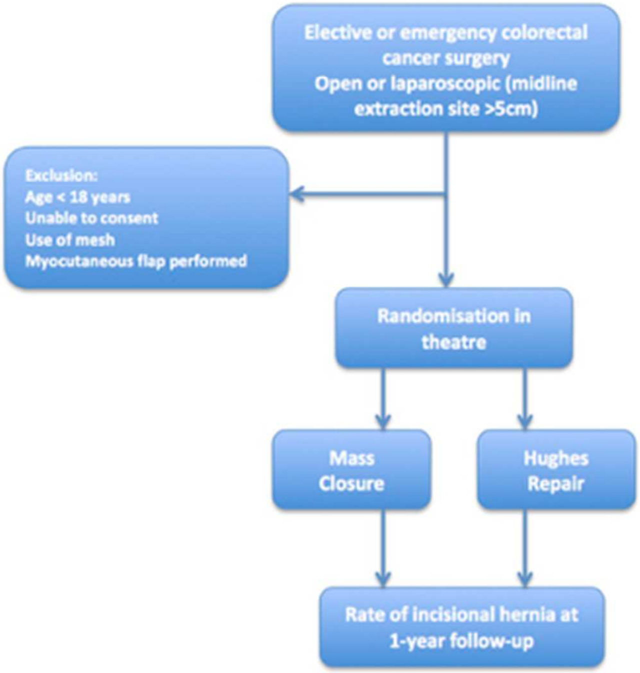

Figure 2. HART Study Design

180x132mm (72 x 72 DPI)

For peer review only - http://bmjopen.bmj.com/site/about/guidelines.xhtml

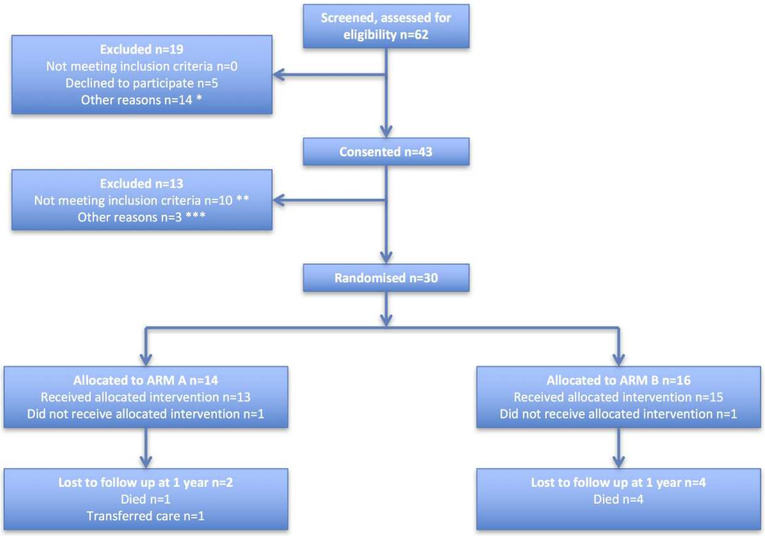

* 9 patients not approached due to staff holidays, 5 patients were screened but not included as feasibility study closed
** 9 patients did not have midline incision >5cm, 1 patient did not have cancer
*** 1 patient was missed in theatre, 2 patients changed their mind before surgery

Figure 3. CONSORT diagram

254x190mm (300 x 300 DPI)

CONSORT 2010 checklist of information to include when reporting a randomised trial*

Section/Topic	Item No	Checklist item	Reported on page No
**Title and abstract**			
	1a	Identification as a randomised trial in the title	1
	1b	Structured summary of trial design, methods, results, and conclusions (for specific guidance see CONSORT for abstracts)	2
**Introduction**			
Background and objectives	2a	Scientific background and explanation of rationale	4-5
	2b	Specific objectives or hypotheses	4-6
**Methods**			
Trial design	3a	Description of trial design (such as parallel, factorial) including allocation ratio	6
	3b	Important changes to methods after trial commencement (such as eligibility criteria), with reasons	n/a
Participants	4a	Eligibility criteria for participants	6-7
	4b	Settings and locations where the data were collected	6-7
Interventions	5	The interventions for each group with sufficient details to allow replication, including how and when they were actually administered	7-8
Outcomes	6a	Completely defined pre-specified primary and secondary outcome measures, including how and when they were assessed	n/a
	6b	Any changes to trial outcomes after the trial commenced, with reasons	n/a
Sample size	7a	How sample size was determined	8
	7b	When applicable, explanation of any interim analyses and stopping guidelines	n/a
Randomisation:			
Sequence generation	8a	Method used to generate the random allocation sequence	7
	8b	Type of randomisation; details of any restriction (such as blocking and block size)	7
Allocation concealment mechanism	9	Mechanism used to implement the random allocation sequence (such as sequentially numbered containers), describing any steps taken to conceal the sequence until interventions were assigned	7
Implementation	10	Who generated the random allocation sequence, who enrolled participants, and who assigned participants to interventions	7
Blinding	11a	If done, who was blinded after assignment to interventions (for example, participants, care providers, those	7

For peer review only - http://bmjopen.bmj.com/site/about/guidelines.xhtml

		assessing outcomes) and how	
	11b	If relevant, description of the similarity of interventions	
Statistical methods	12a	Statistical methods used to compare groups for primary and secondary outcomes	n/a
	12b	Methods for additional analyses, such as subgroup analyses and adjusted analyses	n/a
**Results**			
Participant flow (a diagram is strongly recommended)	13a	For each group, the numbers of participants who were randomly assigned, received intended treatment, and were analysed for the primary outcome	9
	13b	For each group, losses and exclusions after randomisation, together with reasons	9
Recruitment	14a	Dates defining the periods of recruitment and follow-up	9
	14b	Why the trial ended or was stopped	9
Baseline data	15	A table showing baseline demographic and clinical characteristics for each group	14
Numbers analysed	16	For each group, number of participants (denominator) included in each analysis and whether the analysis was by original assigned groups	9
Outcomes and estimation	17a	For each primary and secondary outcome, results for each group, and the estimated effect size and its precision (such as 95% confidence interval)	n/a
	17b	For binary outcomes, presentation of both absolute and relative effect sizes is recommended	n/a
Ancillary analyses	18	Results of any other analyses performed, including subgroup analyses and adjusted analyses, distinguishing pre-specified from exploratory	n/a
Harms	19	All important harms or unintended effects in each group (for specific guidance see CONSORT for harms)	n/a
**Discussion**			
Limitations	20	Trial limitations, addressing sources of potential bias, imprecision, and, if relevant, multiplicity of analyses	10
Generalisability	21	Generalisability (external validity, applicability) of the trial findings	10
Interpretation	22	Interpretation consistent with results, balancing benefits and harms, and considering other relevant evidence	10
**Other information**			
Registration	23	Registration number and name of trial registry	1
Protocol	24	Where the full trial protocol can be accessed, if available	6
Funding	25	Sources of funding and other support (such as supply of drugs), role of funders	8

For peer review only

*We strongly recommend reading this statement in conjunction with the CONSORT 2010 Explanation and Elaboration for important clarifications on all the items. If relevant, we also recommend reading CONSORT extensions for cluster randomised trials, non-inferiority and equivalence trials, non-pharmacological treatments, herbal interventions, and pragmatic trials. Additional extensions are forthcoming: for those and for up to date references relevant to this checklist, see www.consort-statement.org.

For peer review only - http://bmjopen.bmj.com/site/about/guidelines.xhtml

BMJ Open

Hughes Abdominal Repair Trial (HART) - Abdominal wall closure techniques to reduce the incidence of incisional hernias: Feasibility trial for a multi-centre pragmatic randomised controlled trial

Journal:	*BMJ Open*
Manuscript ID	bmjopen-2017-017235.R1
Article Type:	Research
Date Submitted by the Author:	18-Aug-2017
Complete List of Authors:	Harries, Rhiannon; University Hospital of Wales, Department of Colorectal Surgery; Welsh Barbers Research Group<br>Cornish, Julie; Welsh Barbers Research Group; Royal Glamorgan Hospital, Department of Colorectal Surgery<br>Bosanquet, David; University Hospital of Wales, Department of Colorectal Surgery; Welsh Barbers Research Group<br>Rees, Buddug; University Hospital of Wales, Department of Colorectal Surgery<br>Horwood, James; University Hospital of Wales, Department of Colorectal Surgery<br>Islam, Saiful; Swansea University, Trials Unit<br>Bashir, Nadim; Swansea University, Trials Unit<br>Watkins, Alan; Swansea University, Trials Unit<br>Russell, Ian; Swansea University, Trials Unit<br>Torkington, Jared; University Hospital of Wales, Department of Colorectal Surgery
**Primary Subject Heading**:	Surgery
Secondary Subject Heading:	Evidence based practice
Keywords:	incisional hernia, abdominal closure, hughes repair, mass closure, randomised controlled trial, colorectal cancer

SCHOLARONE™
Manuscripts

Hughes Abdominal Repair Trial (HART) - Abdominal wall closure techniques to reduce the incidence of incisional hernias: Feasibility trial for a multi-centre pragmatic randomised controlled trial (ISRCTN 25616490)

Harries RL[1,2], Cornish J[2,3], Bosanquet D[1,2], Rees B[1], Horwood J[1], Islam S[4], Bashir N[4], Watkins A[4], Russell IT[4], Torkington J[1]; on behalf of the HART Trial Management Group

1 University Hospital of Wales, Cardiff

2 Welsh Barbers Research Group

3 Royal Glamorgan Hospital, Llantrisant

4 Swansea Trials Unit, Swansea University

Correspondence should be addressed to:

Prof Jared Torkington, Dep. of Surgery, University Hospital of Wales, Heath Park, Cardiff CF14 4XW

jared.torkington@wales.nhs.uk Tel: +44 2920 747747

For peer review only

Word count: 2240

Article type: Randomised controlled trial

Running title: Hughes Abdominal Repair Trial: Feasibility trial

Conflict of interest: None declared

Keywords: Incisional hernia; Abdominal closure; Hughes repair; Mass closure; randomised controlled trial; colorectal cancer

Abstract

Objectives: Incisional hernias are common complications of midline abdominal closure. The 'Hughes Repair' combines a standard mass closure with a series of horizontal and two vertical mattress sutures within a single suture. There is evidence to suggest this technique is as effective as mesh repair for the operative management of incisional hernias; however, no trials have compared Hughes Repair with standard mass closure for the prevention of incisional hernia formation. This paper aims to test the feasibility of running a randomised controlled trial of a comparison of abdominal wall closure methods following midline incisional surgery for colorectal cancer, in preparation to a definitive randomised controlled trial.

Design and Setting: A feasibility trial (with 1:1 randomisation) conducted perioperatively during colorectal cancer surgery.

Participants: Patients undergoing midline incisional surgery for resection of colorectal cancer.

Interventions: Comparison of two suture techniques (Hughes repair or standard mass closure) for the closure of the midline abdominal wound following surgery for colorectal cancer.

Primary and Secondary Outcomes: A 30-patient feasibility trial assessed recruitment, randomisation, deliverability and early safety of the surgical techniques used.

Results: A total of 30 patients were randomised from 43 patients recruited and consented, over a 5-month period. 14 and 16 patients were randomised to Arm A and B, respectively. There was 1 superficial surgical site infection (SSI) and 2 organ space SSI reported in arm A and 2 superficial SSI and 1 complete wound dehiscence in arm B. There were no suspected unexpected serious adverse reactions reported in either arm. Independent data monitoring committee found no early safety concerns.

Conclusions: The feasibility trial found no early safety concerns and demonstrated that the trial was acceptable to patients. Progression to the pilot and main phases of the trial has now commenced following approval by the independent data monitoring committee.

Trial registration: ISRCTN 25616490

For peer review only

Strengths and Limitations of this study

- The results of this feasibility trial demonstrate that recruitment to a randomised controlled trial comparing suturing techniques in midline incisions following colorectal cancer resection surgery is acceptable to patients with no early safety concerns identified
- This feasibility trial is not powered for a definitive study and simply reports the recruitment, deliverability and safety
- We report blinded outcome data and have not included incisional hernia rates in order to prevent the introduction of bias or reduction of equipoise for future recruitment of the main trial
- We acknowledge that randomising immediately prior to abdominal closure may increase the risk of selection bias into the trial, however to overcome this we have collected information on the reasons why patients were not randomised after consenting in the screening log.
- The setting of the feasibility trial was chosen as the trial's lead site; a high volume teaching hospital. This poses a potential limitation for the main trial at lower volume centres, in terms of ability to recruit participants over the time-period (3 years). However, the sample size required for the pilot and main trial is 800 across roughly 20 sites, which equates to 40 participants required per site and should be achievable over the trial time-period, even for lower volume centres, given the incidence of colorectal cancer in the UK.
- The HART trial is a pragmatic trial and as such we are allowing the control (mass closure) arm to be the responsible consultant surgeon's standard closure technique. We acknowledge that this may introduce a degree of variability in the control arm, but in order to counter this our sample size for the main trial has been powered to be able to stratify for site and surgeon.

For peer review only

Introduction

Incisional hernias are common complications of midline abdominal incisions, with a reported incidence of 12.8% at 2 years follow-up in a systematic review of 14,618 patients [1]. Within patients who have undergone colorectal cancer resectional surgery, the rate of incisional hernia has been reported as high as 39.9%, including both open and laparoscopic approaches (40.9% and 37.1%, respectively) [2]. They can result in significant morbidity, impaired quality of life [3] and frequently require emergency surgery. Despite recent development in mesh technology, incisional hernia repair still has disappointingly high recurrence rates (up to 54% in suture repair and up to 36% in mesh repair [4-5]). Prevention of the development of incisional hernia therefore brings significant benefits for both patients and healthcare provision funding.

'Mass closure' remains the standard technique for abdominal closure (closing all layers of the abdominal wall, excluding the skin), with either non-absorbable or slow-resorbing sutures, such as polydioxanone (PDS) [6]. A systematic review and meta-regression of over 14,000 patients found no difference in incisional hernia rate comparing suture material [1]. This poses the question as to whether improved suture technique may reduce incisional hernia formation. The STITCH trial [7], a Dutch multicentre, randomised controlled trial compared small stitch continuous sutures with large stitch standard mass closure in 560 patients. Results demonstrated a reduction in the rate of incisional hernia from 21% in the large bite group to 13% in the small bite group at one-year follow-up. The CONTINT trial, currently still in recruitment, is comparing continuous with interrupted sutures in closing midline incisions after emergency laparotomy [8].

The European Hernia Society Guidance on the closure of abdominal wall incisions (2015) recommended the use of prophylactic mesh augmentation for an elective midline laparotomy in a high-risk patient in order to reduce the risk of incisional hernia [9]. However, firstly, they determined that the evidence base for this was weak and secondly, in the UK mesh augmentation closure is infrequently used. It is for these reasons that it is still critical for other closure methods to be rigorously assessed for their role in incisional hernia prevention.

The eponymously titled 'Hughes Repair' (Professor Les Hughes, 1932-2011 [10]), also known as the 'far-and-near' or 'Cardiff Repair' [11] combines a standard mass closure (two loop 1 PDS sutures) with a series of horizontal and two vertical mattress sutures within a single suture (1

For peer review only

Nylon); theoretically distributing the load along the incision length as well as across it (**Figure 1**). The principles are:

1. To ensure, by palpation, that only sound normal tissues are used for the repair
2. To use graduated tension for easy approximation
3. Use a monofilament Nylon suture, which has the advantage of slipping easily through tissues to create a pulley system [12].

The Hughes Repair has been shown to have outcomes as effective as the standard mesh repair in incisional hernia repairs [13]. It is also used for closing abdomens when patients are at high risk of incisional hernias, after complete abdominal wound dehiscence and laparostomy [14].

This feasibility trial aimed to establish whether a randomised controlled trial to compare Hughes Repair with standard mass closure for prevention of midline incisional hernia, in patients undergoing colorectal cancer resectional surgery, would be deemed acceptable to patients, achieve adequate recruitment and result in no early safety concerns.

For peer review only

Methods/Design

Study Design

The HART trial hypothesis is that the Hughes Repair will reduce the incidence of clinically detected incisional hernia at one year in patients undergoing midline abdominal wall closure incisions following elective or emergency colorectal cancer surgery when compared with standard mass closure **Figure 2**. This is a 1:1 randomised controlled trial comparing two suture techniques for the closure of the midline abdominal wound following surgery for colorectal cancer. The study was approved by the Wales Research Ethics Committee (MREC 12/WA/0374).

Setting and location

The feasibility trial took place at the trial's lead site University Hospital of Wales, Cardiff; a high-volume teaching hospital (1 of the 20 proposed recruitment sites for the main trial).

Aims and outcome measures

The feasibility trial aimed to assess the ability of the trial to recruit and consent patients over a 5-month period and the deliverability and safety of the Hughes Repair. The acceptability was assessed in terms of percentage of consenting versus refusing participants. Adequacy of recruitment is assessed in terms of number of recruited participants. Operation specific adverse events collected included surgical site infection and full wound dehiscence. Early surgical safety was assessed using post-operative complications, serious adverse event reporting and wound diaries. In this paper we report blinded outcome data and have not included incisional hernia rates in order to prevent the introduction of bias or reduction of equipoise for future recruitment of the main trial.

Independent data monitoring committee reviewed the un-blinded safety data after completion of the feasibility phase. The outcome measures for the full trial have been previously reported [15]; Primary outcome measure being the rate of incisional hernia at 1-year follow-up assessed by clinical examination. The full trial protocol can be accessed via the following link: https://njl-admin.nihr.ac.uk/document/download/2007245

Eligibility Criteria

For peer review only

Eligibility criteria were assessed at two time points; at initial screening and at point of surgical closure /randomisation. Adult patients (aged 18 year or over), able to give informed consent, undergoing either elective colorectal cancer surgery following full staging investigations including an abdominal CT scan or emergency surgery in those with a strong suspicion of colorectal cancer on abdominal CT scan were eligible at point of initial screening. All patients had to be suitable for either Hughes repair or standard mass closure. At point of surgical closure, eligibility was further assessed, and all patients who had a midline incision (open or laparoscopic assisted/ converted) of 5cm or more in length were deemed suitable for randomisation. Patients requiring mesh insertion or having an abdominal musculofascial flap for closure of the perineal defect in abdomino-perineal wound closure were excluded.

Consent

Patients were identified, approached and provided with a patient information leaflet. Consent for trial participation, was gained by either consultant surgeons or surgical registrars who had current 'Good Clinical Practice' certification.

Randomisation and Data Collection

An adaptive randomisation design was used to allocate eligible patients to groups of similar size [16]. Patients were randomised in a 1:1 ratio to either Mass closure or Hughes Repair. Randomisation took place during surgery and as close as possible to the time when the surgeon commenced closure. During the feasibility trial, a telephone randomisation system was used. The patient was blinded to the treatment allocation assigned to them. Data management was supported by the Swansea Trials Unit.

Surgical Quality Assurance

To assure the quality of the repair techniques, all surgeons participating in the trial (consultants and registrars) completed training and quality assessment on the Hughes Repair. All participating surgeons were assessed by the Chief Investigator and were approved only when closure technique was satisfactory. A reference instructional video was provided to participating surgeons. To monitor the training of professionals contributing to HART, a log was maintained with details of training, both surgical and in research governance notably

For peer review only

'Good Clinical Practice'. For the purposes of this pragmatic study, mass closure was taken to be the responsible consultant surgeon's standard closure technique.

Radiological Evaluation of incisional hernia

A dedicated trial radiologist determined whether there was a hernia present on the 1-year colorectal cancer surveillance CT scan. They defined an incisional hernia as herniation of the bowel or other intra-abdominal content outside the abdominal wall, and also identified the presence of other hernias and the quality of the recti muscle. All scans were performed using the standard departmental protocol for follow-up scans.

Sample size

The feasibility trial aimed to recruit a total of 30 patients over a 5-month period. The sample size for the main study has been published previously [14].

Funding

The NIHR Health Technology Assessment programme requested this feasibility trial as a prerequisite for awarding grant 12/35/29 for the pilot and main phases of HART.

Adverse events

An adverse event (AE) was defined as any untoward medical occurrence in a clinical trial participant to whom a study intervention has been administered and which does not necessarily have a causal relationship with this treatment. An AE can therefore be any unfavourable and unintended sign (including abnormal laboratory finding), symptom or disease. The following listed AEs that are considered expected for patients undergoing colorectal surgery: lower respiratory tract infection, urinary tract infection, anastomotic leak, intra-abdominal sepsis, deep vein thrombosis, pulmonary embolus, wound infection, surgical site infection, wound breakdown, paralytic ileus, bleeding, myocardial infarction, and stoma complications (prolapsed, retraction, dehiscence or hernia). However, if these events lead to death, that would be considered unexpected. These events may be classified as serious and will be recorded as such but will not require reporting to Research Ethics Committee. Additional information may be requested for adverse events of special interest such as wound breakdown and surgical site infections.

For peer review only

A serious adverse event (SAE) is an adverse event which results in any of the following: death, was life-threatening, required hospitalization or prolongation of existing hospitalization, persistent or significant disability or incapacity, consists of a congenital anomaly or birth defect, or is otherwise considered medically significant by the investigator.

Statistical analysis

A two-tailed fisher's exact test was used to compare SAE rate between both arms. Differences were considered to be statistically significant at $p \leq 0.05$.

For peer review only

Results

Recruitment and randomisation

A total of 62 patients were screened and assessed for eligibility for entry into the trial over a 5-month time period, October 2013 to February 2014. (**Figure 3**). Of those screened, 43 patients consented to entry into the trial (69%). A total of 30 of the 43 patients were randomised in the operating theatre (14 patients were randomised to arm A and 16 patients were randomised to arm B). The reasons for exclusion are described in the CONSORT diagram (**Figure 3**).

For the 30 patients who were randomised, the median age was 74 years (IQR 66-78). There were 23 males and 7 females. Demographical data is presented in **Table 1**.

For peer review only

Table 1. Patient demographics and clinical characteristics

	**Arm A** (N=14)	**Arm B** (N=16)	**Total** (N=30)
Gender			
Male	10 (71%)	13 (81%)	23 (77%)
Female	4 (29%)	3 (19%)	7 (23%)
Median Age (IQR)	75 (61-78)	73 (68- 77)	74 (66-78)
Mean BMI (Min-Max)	30 (22-49)	29 (18-42)	29 (18-49)
Smoker	1 (7%)	3 (19%)	4 (13%)
Steroid/ immunosuppression use	0 (0%)	0 (0%)	0 (0%)
Diabetes	5 (36%)	4 (25%)	9 (30%)
Connective tissue disorder	1 (7%)	0 (0%)	1 (3%)
COPD	1 (7%)	2 (13%)	3 (10%)
AAA (known or previous repair)	0 (0%)	0 (0%)	0 (0%)
Previous abdominal surgery	8 (57%)	8 (50%)	15 (50%)
Neoadjuvant chemotherapy	1 (7%)	0 (0%)	1 (3%)
Neoadjuvant radiotherapy	1 (7%)	1 (6%)	2 (7%)
Incisional Hernia present pre-operatively	0 (0%)	1 (6%)	1 (3%)
Previous incisional hernia repair	1 (7%)	0 (0%)	1 (3%)
Non incisional hernia present pre-operatively	0 (0%)	3 (19%)	3 (10%)
Mode of surgery			
Laparoscopic	4 (27%)	6 (38%)	10 (33%)
Laparoscopic converted	7 (50%)	3 (19%)	10 (33%)
Open	3 (21%)	7 (44%)	10 (33%)

For peer review only

In arm A, one patient had died prior to 12-month follow-up and a further patient had transferred care to another unit. In arm B, one patient had died prior to 12-month follow-up.

Safety data

There were a total of 16 serious adverse events reported in 10 patients (**Table 2**);

Table 2. Reported serious adverse events

	**Arm A**	**Arm B**
Myocardial infarction	2	2
Lower respiratory tract infection	2	1
Pulmonary embolism	1	0
Renal failure	0	1
Anastomotic leak	2	0
Parastomal hernia	0	1
Superficial surgical site infection	2	0
Dehiscence	0	1
Death*	1	0
**Total SAEs**	**10**	**6**
**Total Patients affected**	**5**	**5**

* 1 SAE reported was reported as 'death', therefore it has had to be listed as an event of death. There were two other SAEs that resulted in death within the feasibility study

serious adverse event rate was 34% in arm A and 31% in arm B (p=1.0000). There were no suspected unexpected serious adverse reactions reported in either arm. With regard to wound related complications, there was 1 superficial surgical site infection and 2 organ space surgical site infections reported in arm A and 2 superficial surgical site infections and 1 complete wound dehiscence requiring a return to theatre in arm B (**Table 3**).

For peer review only

Table 3. Wound related complications

	Arm A	Arm B
Superficial SSI	1	2
Deep SSI	0	0
Organ space SSI	2	0
Wound dehiscence	0	1
**Total wound related complications**	**3**	**3**

The Data Monitoring Committee (DMC) reviewed the un-blinded adverse events data and identified no safety concerns.

For peer review only

Discussion

The results of this feasibility trial demonstrated that a randomised controlled trial designed to compare two suture techniques for the prevention of midline incisional hernia, in patients undergoing cancer resectional surgery, was able to recruit 30 patients over 5 months, as planned. This suggests that the proposed sample size of 800 patients for the main full trial is achievable in the time scale with the proposed number of sites recruiting (approximately 20) [14].

The feasibility trial results established that the trial was acceptable to patients. Patient participation rates were high, demonstrated by 69% of all eligible patients consenting to participation in the trial. Nine patients were screened for eligibility but not consented due to staff shortages highlighting the importance of having adequate number of approved consenting staff on the delegation log. Due to the nature of the study it was accepted that not all patients consented would eventually be randomised. In fact, there was a higher than expected number of patients consented and not eventually randomised (31%). Patients were consented but not randomised if the intraoperative procedure performed did not meet the inclusion criteria, for example not midline incision, conversion to open procedure using a non-midline incision, or emergency patients found not to have a tumour intra-operatively. We acknowledge that this method may increase the risk of selection bias into the trial, however to overcome this we have collected information on the reasons why patients were not randomised after consenting (**Figure 3**).

The setting of the feasibility trial was chosen as the trial's lead site; a high volume teaching hospital. This poses a potential limitation for the main trial at lower volume centres, in terms of ability to recruit participants over the time-period (3 years). However, the sample size required for the pilot and main trial is 800 across roughly 20 sites, which equates to 40 participants required per site and should be achievable over the trial time-period, even for lower volume centres, particularly given the incidence of colorectal cancer within the UK.

The HART trial is a pragmatic trial and as such we are allowing the control (mass closure) arm to be the responsible consultant surgeon's standard closure technique. We acknowledge that this may introduce a degree of variability in the control arm, but in order to counter this our sample size for the main trial has been powered to be able to stratify for site and surgeon.

For peer review only

The serious adverse event rate and wound related complications were similar between both arms and reassuringly there were no suspected unexpected serious adverse reactions reported. It is anticipated that reporting on the full trial will take place in 2019.

For peer review only

List of abbreviations

AAA: Abdominal aortic aneurysm

BMI: Body Mass Index

CONTINT: CONTinuous versus INTerrupted abdominal wall closure after emergency midline laparotomy

COPD: Chronic Obstructive Pulmonary Disease

CT: Computed tomography

DMC: Data Monitoring Committee

HART: Hughes Abdominal Repair Trial

IQR: interquartile range

NIHR: National Institute of Health Research

PDS: polydioxanone

SAEs: Serious Adverse Events

SSI: Surgical Site Infection

STITCH: Suture Techniques to reduce the Incidence of The inCisional Hernia (RCT)

Competing Interests

There are no competing interests to declare

Authors Contributions

RLH, JC, DB, BR, ITR, JT conceived, designed and drafted the initial protocol

JH aided in redrafting and revising the protocol and contributed heavily to completion of the feasibility trial and pilot stages

SI, AW, NB, ITR have written and designed the analysis plan and revised the protocol

JC, DB, ITR and JT led the team that acquired the funding

RLH wrote the first draft of the manuscript

All authors agree to be accountable for all aspects of the work. All authors read and approved the final manuscript.

For peer review only

References

1. Bosanquet D, Ansell J, Abdelrahman T, Cornish J, Harries R, Stimpson A, Davies L, Glasbey J, Frewer K, Frewer N, Russell D, Russell I, Torkington J. Systematic Review and Meta-Regression of factors affecting midline incisional hernia rates: Analysis of 14,618 patients. PLoS ONE 2015;10(9): e0138745.

2. Pereira JA, Pera M, Grande L. [Incidence of incisional hernia after open and laparoscopic colorectal cancer resection]. Cir Esp. 2013;91(1):44-9.

3. van Ramshorst GH, Eker HH, Hop WC, Jeekel J, Lange JF. Impact of incisional hernia on health-related quality of life and body image: a prospective cohort study. Am J Surg, 2012;204(2):144-50.

4. Diener MK, Voss S, Jensen K, Büchler MW, Seiler CM. Elective midline laparotomy closure: the INLINE systematic review and meta-analysis. Ann Surg, 2010; 251(5):843-56.

5. van 't Riet M, Steyerberg EW, Nellensteyn J, Bonjer HJ, Jeekel J. Meta-analysis of techniques for closure of midline abdominal incisions. Br J Surg, 2002;89(11):1350-6.

6. Sajid MS, Parampalli U, Baig MK, McFall MR. A systematic review on the effectiveness of slowly-absorbable versus non-absorbable sutures for abdominal fascial closure following laparotomy. Int J Surg, 2011; 9(8):615-25.

7. Deerenberg EB, Harlaar JJ, Steyerberg EW, Lont HE, van Doorn HC, Heisterkamp J, Wijnhoven BP, Schouten WR, Cense HA, Stockmann HB, Berends FJ, Dijkhuizen FP, Dwarkasing RS, Jairam AP, van Ramshorst GH, Kleinrensink GJ, Jeekel J, Lange JF. Small bites versus large bites for closure of abdominal midline incisions (STITCH): a double-blind, multicentre, randomised controlled trial. Lancet. 2015, 386(10000):1254-60.

8. Rahbari NN, Knebel P, Kieser M, Bruckner T, Bartsch DK, Friess H, Mihaljevic AL, Stern J, Diener MK, Voss S, Rossion I, Büchler MW, Seiler CM. Design and current status of CONTINT: continuous versus interrupted abdominal wall closure after emergency midline laparotomy - a randomized controlled multicenter trial [NCT00544583]. Trials, 2012;13:72.

For peer review only

9. Muysoms FE, Antoniou SA, Bury K, Campanelli G, Conze J, Cuccurullo D, Beaux AC, Deerenberg EB, East B, Fortelny RH, Gillion J, Henriksen NA, Israelsson L, Jairam A, Jänes A, Jeekel J, López-Cano M, Miserez M, Morales-Conde S, Sanders DL, Simons MP, Śmietański M, Venclauskas L, Berrevoet F; EuropeanHernia Society. European Hernia Society guidelines on the closure of abdominal wall incisions. Hernia 2015;19:1–24.

10. Hughes BR & Webster D. Leslie Ernest Hughes OBITUARY. BMJ, 2011. 343.

11. Shukla VK, Gupta A, Singh H, Pandey M, Gautam A. Cardiff repair of incisional hernia: a university hospital experience. Eur J Surg, 1998;164(4):271-4.

12. Mudge M, Harding KG, Hughes LE. Incisional hernia. Br J Surg, 1986;73(1): 82.

13. Godara R, Garg P, Shankar G. Comparative evaluation of Cardiff repair and mesh plasty in incisional hernias. Internet J Surg [Internet]. 2007; 9 [about 8 p.].

14. Malik R & Scott NA. Double near and far prolene suture closure: a technique for abdominal wall closure after laparostomy. Br J Surg, 2001;88(1):146-7.

15. Cornish J, Harries RL, Bosanquet D, Rees B, Ansell J, Frewer N, Dhruva Rao P, Parry C, Ellis-Owen R, Phillips SM, Morris C, Horwood J, Davies ML, Davies MM, Hargest R, Hilton J, Harris D, Ben-Sassi A, Rajagopal R, Hanratty D, Islam S, Watkins A, Bashir N, Jones S, Russell IR, Torkington J, on behalf of the HART Trial Management Group. Hughes Abdominal Repair Trial (HART) - Abdominal wall closure techniques to reduce the incidence of incisional hernias: Study protocol for a multi-centre pragmatic randomised controlled trial (ISRCTN 25616490). Trials 2016; 17(1):454.

16. Russell D, Hoare ZSJ, Whitaker Rh, Whitaker CJ, Russell IT. Generalised method for adaptive randomisation in clinical trials. Statistics in Medicine 2011; 30: 922-34.

For peer review only

For peer review only

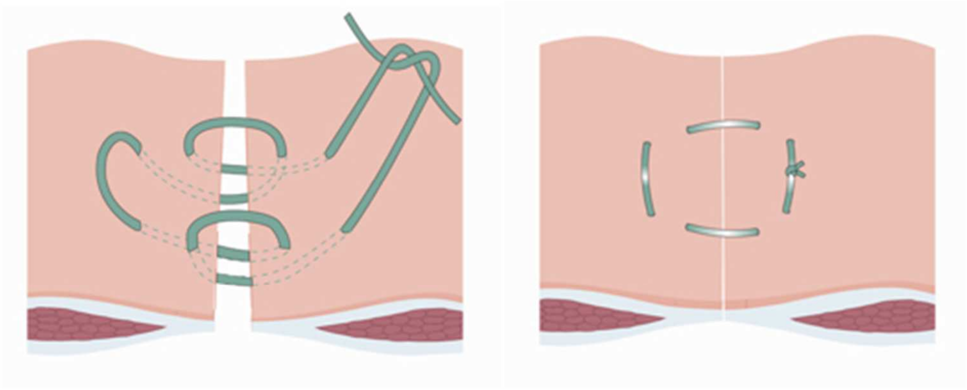

Figure 1. Diagram showing the Hughes closure method, using a combination of standard mass closure with a series of horizontal and two vertical mattress sutures within a single suture. When the sutures are pulled to close the defect, the sutures lie both across and along the incision.

191x79mm (72 x 72 DPI)

For peer review only

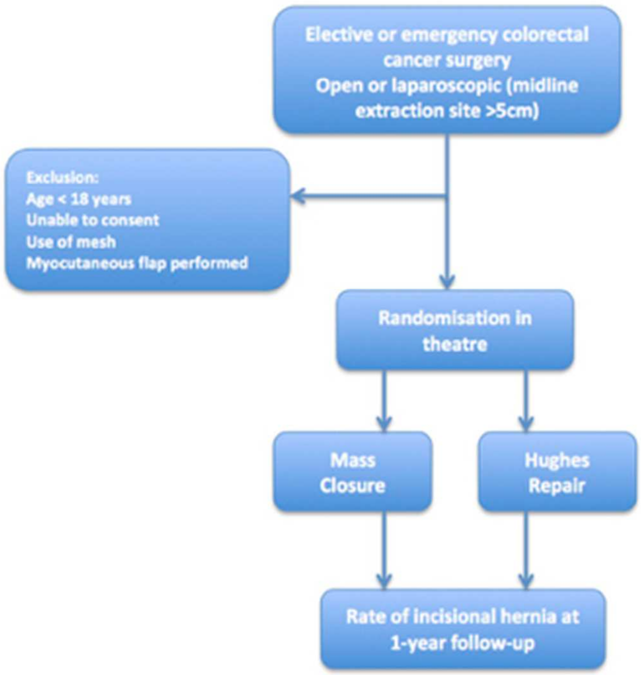

Figure 2. HART Study Design

180x132mm (72 x 72 DPI)

For peer review only

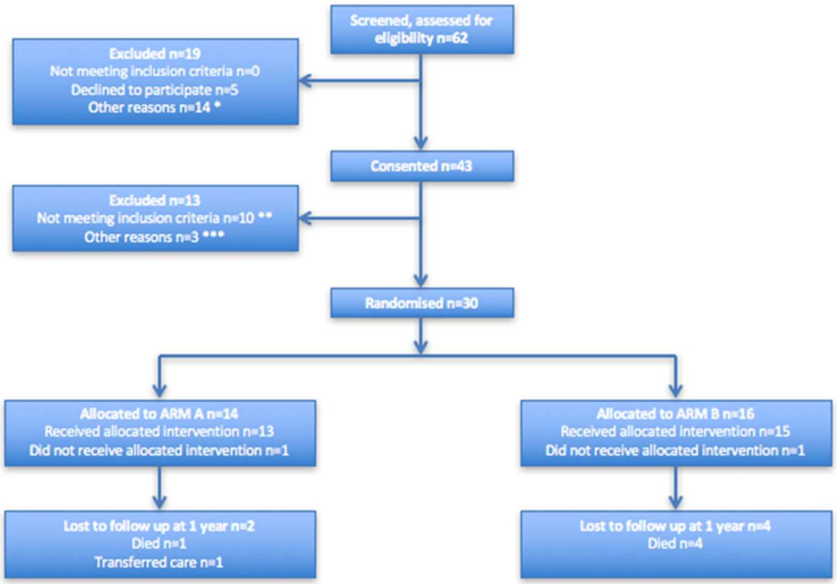

* 9 patients not approached due to staff absence, 5 patients were screened but not included as feasibility study closed
** 9 patients did not have midline incision >5cm, 1 patient did not have cancer
*** 1 patient was missed in theatre, 2 patients changed their mind before surgery

Figure 3. CONSORT diagram‼ ┼

222x164mm (72 x 72 DPI)

For peer review only - http://bmjopen.bmj.com/site/about/guidelines.xhtml

CONSORT 2010 checklist of information to include when reporting a randomised trial*

Section/Topic	Item No	Checklist item	Reported on page No
**Title and abstract**			
	1a	Identification as a randomised trial in the title	1
	1b	Structured summary of trial design, methods, results, and conclusions (for specific guidance see CONSORT for abstracts)	2
**Introduction**			
Background and objectives	2a	Scientific background and explanation of rationale	4-5
	2b	Specific objectives or hypotheses	4-6
**Methods**			
Trial design	3a	Description of trial design (such as parallel, factorial) including allocation ratio	6
	3b	Important changes to methods after trial commencement (such as eligibility criteria), with reasons	n/a
Participants	4a	Eligibility criteria for participants	6-7
	4b	Settings and locations where the data were collected	6-7
Interventions	5	The interventions for each group with sufficient details to allow replication, including how and when they were actually administered	7-8
Outcomes	6a	Completely defined pre-specified primary and secondary outcome measures, including how and when they were assessed	n/a
	6b	Any changes to trial outcomes after the trial commenced, with reasons	n/a
Sample size	7a	How sample size was determined	8
	7b	When applicable, explanation of any interim analyses and stopping guidelines	n/a
Randomisation:			
Sequence generation	8a	Method used to generate the random allocation sequence	7
	8b	Type of randomisation; details of any restriction (such as blocking and block size)	7
Allocation concealment mechanism	9	Mechanism used to implement the random allocation sequence (such as sequentially numbered containers), describing any steps taken to conceal the sequence until interventions were assigned	7
Implementation	10	Who generated the random allocation sequence, who enrolled participants, and who assigned participants to interventions	7
Blinding	11a	If done, who was blinded after assignment to interventions (for example, participants, care providers, those	7

For peer review only - http://bmjopen.bmj.com/site/about/guidelines.xhtml

		assessing outcomes) and how	
	11b	If relevant, description of the similarity of interventions	
Statistical methods	12a	Statistical methods used to compare groups for primary and secondary outcomes	n/a
	12b	Methods for additional analyses, such as subgroup analyses and adjusted analyses	n/a
**Results**			
Participant flow (a diagram is strongly recommended)	13a	For each group, the numbers of participants who were randomly assigned, received intended treatment, and were analysed for the primary outcome	9
	13b	For each group, losses and exclusions after randomisation, together with reasons	9
Recruitment	14a	Dates defining the periods of recruitment and follow-up	9
	14b	Why the trial ended or was stopped	9
Baseline data	15	A table showing baseline demographic and clinical characteristics for each group	14
Numbers analysed	16	For each group, number of participants (denominator) included in each analysis and whether the analysis was by original assigned groups	9
Outcomes and estimation	17a	For each primary and secondary outcome, results for each group, and the estimated effect size and its precision (such as 95% confidence interval)	n/a
	17b	For binary outcomes, presentation of both absolute and relative effect sizes is recommended	n/a
Ancillary analyses	18	Results of any other analyses performed, including subgroup analyses and adjusted analyses, distinguishing pre-specified from exploratory	n/a
Harms	19	All important harms or unintended effects in each group (for specific guidance see CONSORT for harms)	n/a
**Discussion**			
Limitations	20	Trial limitations, addressing sources of potential bias, imprecision, and, if relevant, multiplicity of analyses	10
Generalisability	21	Generalisability (external validity, applicability) of the trial findings	10
Interpretation	22	Interpretation consistent with results, balancing benefits and harms, and considering other relevant evidence	10
**Other information**			
Registration	23	Registration number and name of trial registry	1
Protocol	24	Where the full trial protocol can be accessed, if available	6
Funding	25	Sources of funding and other support (such as supply of drugs), role of funders	8

*We strongly recommend reading this statement in conjunction with the CONSORT 2010 Explanation and Elaboration for important clarifications on all the items. If relevant, we also recommend reading CONSORT extensions for cluster randomised trials, non-inferiority and equivalence trials, non-pharmacological treatments, herbal interventions, and pragmatic trials. Additional extensions are forthcoming: for those and for up to date references relevant to this checklist, see www.consort-statement.org.

For peer review only

For peer review only - http://bmjopen.bmj.com/site/about/guidelines.xhtml

BMJ Open

Hughes Abdominal Repair Trial (HART) - Abdominal wall closure techniques to reduce the incidence of incisional hernias: Feasibility trial for a multi-centre pragmatic randomised controlled trial

Journal:	*BMJ Open*
Manuscript ID	bmjopen-2017-017235.R2
Article Type:	Research
Date Submitted by the Author:	16-Sep-2017

Complete List of Authors:	Harries, Rhiannon; University Hospital of Wales, Department of Colorectal Surgery; Welsh Barbers Research Group<br>Cornish, Julie; Welsh Barbers Research Group; Royal Glamorgan Hospital, Department of Colorectal Surgery<br>Bosanquet, David; University Hospital of Wales, Department of Colorectal Surgery; Welsh Barbers Research Group<br>Rees, Buddug; University Hospital of Wales, Department of Colorectal Surgery<br>Horwood, James; University Hospital of Wales, Department of Colorectal Surgery<br>Islam, Saiful; Swansea University, Trials Unit<br>Bashir, Nadim; Swansea University, Trials Unit<br>Watkins, Alan; Swansea University, Trials Unit<br>Russell, Ian; Swansea University, Trials Unit<br>Torkington, Jared; University Hospital of Wales, Department of Colorectal Surgery

**Primary Subject Heading**:	Surgery
Secondary Subject Heading:	Evidence based practice
Keywords:	incisional hernia, abdominal closure, hughes repair, mass closure, randomised controlled trial, colorectal cancer

SCHOLARONE™
Manuscripts

Hughes Abdominal Repair Trial (HART) - Abdominal wall closure techniques to reduce the incidence of incisional hernias: Feasibility trial for a multi-centre pragmatic randomised controlled trial (ISRCTN 25616490)

Harries RL[1,2], Cornish J[2,3], Bosanquet D[1,2], Rees B[1], Horwood J[1], Islam S[4], Bashir N[4], Watkins A[4], Russell IT[4], Torkington J[1]; on behalf of the HART Trial Management Group

1 University Hospital of Wales, Cardiff

2 Welsh Barbers Research Group

3 Royal Glamorgan Hospital, Llantrisant

4 Swansea Trials Unit, Swansea University

Correspondence should be addressed to:

Prof Jared Torkington, Dep. of Surgery, University Hospital of Wales, Heath Park, Cardiff CF14 4XW

jared.torkington@wales.nhs.uk Tel: +44 2920 747747

For peer review only

Word count: 2240

Article type: Randomised controlled trial

Running title: Hughes Abdominal Repair Trial: Feasibility trial

Conflict of interest: None declared

Keywords: Incisional hernia; Abdominal closure; Hughes repair; Mass closure; randomised controlled trial; colorectal cancer

Abstract

Objectives: Incisional hernias are common complications of midline abdominal closure. The 'Hughes Repair' combines a standard mass closure with a series of horizontal and two vertical mattress sutures within a single suture. There is evidence to suggest this technique is as effective as mesh repair for the operative management of incisional hernias; however, no trials have compared Hughes Repair with standard mass closure for the prevention of incisional hernia formation. This paper aims to test the feasibility of running a randomised controlled trial of a comparison of abdominal wall closure methods following midline incisional surgery for colorectal cancer, in preparation to a definitive randomised controlled trial.

Design and Setting: A feasibility trial (with 1:1 randomisation) conducted perioperatively during colorectal cancer surgery.

Participants: Patients undergoing midline incisional surgery for resection of colorectal cancer.

Interventions: Comparison of two suture techniques (Hughes repair or standard mass closure) for the closure of the midline abdominal wound following surgery for colorectal cancer.

Primary and Secondary Outcomes: A 30-patient feasibility trial assessed recruitment, randomisation, deliverability and early safety of the surgical techniques used.

Results: A total of 30 patients were randomised from 43 patients recruited and consented, over a 5-month period. 14 and 16 patients were randomised to Arm A and B, respectively. There was 1 superficial surgical site infection (SSI) and 2 organ space SSI reported in arm A and 2 superficial SSI and 1 complete wound dehiscence in arm B. There were no suspected unexpected serious adverse reactions reported in either arm. Independent data monitoring committee found no early safety concerns.

Conclusions: The feasibility trial found no early safety concerns and demonstrated that the trial was acceptable to patients. Progression to the pilot and main phases of the trial has now commenced following approval by the independent data monitoring committee.

Trial registration: ISRCTN 25616490

For peer review only

Strengths and Limitations of this study

- This feasibility trial is not powered for a definitive study and simply reports the recruitment, deliverability and safety
- We report blinded outcome data and have not included incisional hernia rates in order to prevent the introduction of bias or reduction of equipoise for future recruitment of the main trial
- We acknowledge that randomising immediately prior to abdominal closure may increase the risk of selection bias into the trial, however to overcome this we have collected information on the reasons why patients were not randomised after consenting in the screening log.
- The setting of the feasibility trial was chosen as the trial's lead site; a high volume teaching hospital. This poses a potential limitation for the main trial at lower volume centres, in terms of ability to recruit participants over the time-period (3 years).
- The HART trial is a pragmatic trial and as such we are allowing the control (mass closure) arm to be the responsible consultant surgeon's standard closure technique, and acknowledge that this may introduce a degree of variability in the control arm.

For peer review only

Introduction

Incisional hernias are common complications of midline abdominal incisions, with a reported incidence of 12.8% at 2 years follow-up in a systematic review of 14,618 patients [1]. Within patients who have undergone colorectal cancer resectional surgery, the rate of incisional hernia has been reported as high as 39.9%, including both open and laparoscopic approaches (40.9% and 37.1%, respectively) [2]. They can result in significant morbidity, impaired quality of life [3] and frequently require emergency surgery. Despite recent development in mesh technology, incisional hernia repair still has disappointingly high recurrence rates (up to 54% in suture repair and up to 36% in mesh repair [4-5]). Prevention of the development of incisional hernia therefore brings significant benefits for both patients and healthcare provision funding.

'Mass closure' remains the standard technique for abdominal closure (closing all layers of the abdominal wall, excluding the skin), with either non-absorbable or slow-resorbing sutures, such as polydioxanone (PDS) [6]. A systematic review and meta-regression of over 14,000 patients found no difference in incisional hernia rate comparing suture material [1]. This poses the question as to whether improved suture technique may reduce incisional hernia formation. The STITCH trial [7], a Dutch multicentre, randomised controlled trial compared small stitch continuous sutures with large stitch standard mass closure in 560 patients. Results demonstrated a reduction in the rate of incisional hernia from 21% in the large bite group to 13% in the small bite group at one-year follow-up. The CONTINT trial, currently still in recruitment, is comparing continuous with interrupted sutures in closing midline incisions after emergency laparotomy [8].

The European Hernia Society Guidance on the closure of abdominal wall incisions (2015) recommended the use of prophylactic mesh augmentation for an elective midline laparotomy in a high-risk patient in order to reduce the risk of incisional hernia [9]. However, firstly, they determined that the evidence base for this was weak and secondly, in the UK mesh augmentation closure is infrequently used. It is for these reasons that it is still critical for other closure methods to be rigorously assessed for their role in incisional hernia prevention.

The eponymously titled 'Hughes Repair' (Professor Les Hughes, 1932-2011 [10]), also known as the 'far-and-near' or 'Cardiff Repair' [11] combines a standard mass closure (two loop 1 PDS sutures) with a series of horizontal and two vertical mattress sutures within a single suture (1

For peer review only

Nylon); theoretically distributing the load along the incision length as well as across it (**Figure 1**). The principles are:

1. To ensure, by palpation, that only sound normal tissues are used for the repair
2. To use graduated tension for easy approximation
3. Use a monofilament Nylon suture, which has the advantage of slipping easily through tissues to create a pulley system [12].

The Hughes Repair has been shown to have outcomes as effective as the standard mesh repair in incisional hernia repairs [13]. It is also used for closing abdomens when patients are at high risk of incisional hernias, after complete abdominal wound dehiscence and laparostomy [14].

This feasibility trial aimed to establish whether a randomised controlled trial to compare Hughes Repair with standard mass closure for prevention of midline incisional hernia, in patients undergoing colorectal cancer resectional surgery, would be deemed acceptable to patients, achieve adequate recruitment and result in no early safety concerns.

For peer review only

Methods/Design

Study Design

The HART trial hypothesis is that the Hughes Repair will reduce the incidence of clinically detected incisional hernia at one year in patients undergoing midline abdominal wall closure incisions following elective or emergency colorectal cancer surgery when compared with standard mass closure **Figure 2**. This is a 1:1 randomised controlled trial comparing two suture techniques for the closure of the midline abdominal wound following surgery for colorectal cancer. The study was approved by the Wales Research Ethics Committee (MREC 12/WA/0374).

Setting and location

The feasibility trial took place at the trial's lead site University Hospital of Wales, Cardiff; a high-volume teaching hospital (1 of the 20 proposed recruitment sites for the main trial).

Aims and outcome measures

The feasibility trial aimed to assess the ability of the trial to recruit and consent patients over a 5-month period and the deliverability and safety of the Hughes Repair. The acceptability was assessed in terms of percentage of consenting versus refusing participants. Adequacy of recruitment is assessed in terms of number of recruited participants. Operation specific adverse events collected included surgical site infection and full wound dehiscence. Early surgical safety was assessed in terms of serious event and wound complication rates. In this paper we report blinded outcome data and have not included incisional hernia rates in order to prevent the introduction of bias or reduction of equipoise for future recruitment of the main trial.

Independent data monitoring committee reviewed the un-blinded safety data after completion of the feasibility phase. The outcome measures for the full trial have been previously reported [15]; Primary outcome measure being the rate of incisional hernia at 1-year follow-up assessed by clinical examination. The full trial protocol can be accessed via the following link: https://njl-admin.nihr.ac.uk/document/download/2007245. Follow-up will continue for 5 years post-operatively, however in this paper, only 12-month lost to follow-up

For peer review only

data will be presented as the aim of this feasibility trial is to assess the deliverability and safety of the trial.

Eligibility Criteria

Eligibility criteria were assessed at two time points; at initial screening and at point of surgical closure /randomisation. Adult patients (aged 18 year or over), able to give informed consent, undergoing either elective colorectal cancer surgery following full staging investigations including an abdominal CT scan or emergency surgery in those with a strong suspicion of colorectal cancer on abdominal CT scan were eligible at point of initial screening. All patients had to be suitable for either Hughes repair or standard mass closure. At point of surgical closure, eligibility was further assessed, and all patients who had a midline incision (open or laparoscopic assisted/ converted) of 5cm or more in length were deemed suitable for randomisation. Patients requiring mesh insertion or having an abdominal musculofascial flap for closure of the perineal defect in abdomino-perineal wound closure were excluded.

Consent

Patients were identified, approached and provided with a patient information leaflet. Consent for trial participation, was gained by either consultant surgeons or surgical registrars who had current 'Good Clinical Practice' certification.

Randomisation and Data Collection

An adaptive randomisation design was used to allocate eligible patients to groups of similar size; This randomisation is based on an independent, computer-based sequence, generated from an implementation of the dynamic algorithm, using operation category (elective or emergency) and surgeon as stratifying variables [16]. Patients were randomised in a 1:1 ratio to either Mass closure or Hughes Repair. Randomisation took place during surgery and as close as possible to the time when the surgeon commenced closure. During the feasibility trial, a telephone randomisation system was used. The patient was blinded to the treatment allocation assigned to them. Data management was supported by the Swansea Trials Unit.

Surgical Quality Assurance

For peer review only

To assure the quality of the repair techniques, all surgeons participating in the trial (consultants and registrars) completed training and quality assessment on the Hughes Repair. All participating surgeons were assessed by the Chief Investigator and were approved only when closure technique was satisfactory. A reference instructional video was provided to participating surgeons. To monitor the training of professionals contributing to HART, a log was maintained with details of training, both surgical and in research governance notably 'Good Clinical Practice'. For the purposes of this pragmatic study, mass closure was taken to be the responsible consultant surgeon's standard closure technique.

Radiological Evaluation of incisional hernia

A dedicated trial radiologist determined whether there was a hernia present on the 1-year colorectal cancer surveillance CT scan. They defined an incisional hernia as herniation of the bowel or other intra-abdominal content outside the abdominal wall, and also identified the presence of other hernias and the quality of the recti muscle. All scans were performed using the standard departmental protocol for follow-up scans.

Sample size

The feasibility trial aimed to recruit a total of 30 patients over a 5-month period, because the HART trial management group felt that such a sample size was indicative of the ability to recruit the sample proposed for the main trial within the established time frame. The sample size for the main study has been published previously [14].

Funding

The NIHR Health Technology Assessment programme requested this feasibility trial as a prerequisite for awarding grant 12/35/29 for the pilot and main phases of HART.

Adverse events

An adverse event (AE) was defined as any untoward medical occurrence in a clinical trial participant to whom a study intervention has been administered and which does not necessarily have a causal relationship with this treatment. An AE can therefore be any unfavourable and unintended sign (including abnormal laboratory finding), symptom or disease. The following listed AEs that are considered expected for patients undergoing

For peer review only

colorectal surgery: lower respiratory tract infection, urinary tract infection, anastomotic leak, intra-abdominal sepsis, deep vein thrombosis, pulmonary embolus, wound infection, surgical site infection, wound breakdown, paralytic ileus, bleeding, myocardial infarction, and stoma complications (prolapsed, retraction, dehiscence or hernia). However, if these events lead to death, that would be considered unexpected. These events may be classified as serious and will be recorded as such but will not require reporting to Research Ethics Committee. Additional information may be requested for adverse events of special interest such as wound breakdown and surgical site infections.

A serious adverse event (SAE) is an adverse event which results in any of the following: death, was life-threatening, required hospitalization or prolongation of existing hospitalization, persistent or significant disability or incapacity, consists of a congenital anomaly or birth defect, or is otherwise considered medically significant by the investigator.

Statistical analysis

A two-tailed fisher's exact test was used to compare SAE rate between both arms. Differences were considered to be statistically significant at $p \leq 0.05$.

For peer review only

Results

Recruitment and randomisation

A total of 62 patients were screened and assessed for eligibility for entry into the trial over a 5-month time period, October 2013 to February 2014. (**Figure 3**). Of those screened, 43 patients consented to entry into the trial (69%). A total of 30 of the 43 patients were randomised in the operating theatre (14 patients were randomised to arm A and 16 patients were randomised to arm B). The reasons for exclusion are described in the CONSORT diagram (**Figure 3**).

For the 30 patients who were randomised, the median age was 74 years (IQR 66-78). There were 23 males and 7 females. Demographical data is presented in **Table 1**.

For peer review only

Table 1. Patient demographics and clinical characteristics

	**Arm A** (N=14)	**Arm B** (N=16)	**Total** (N=30)
Gender			
Male	10 (71%)	13 (81%)	23 (77%)
Female	4 (29%)	3 (19%)	7 (23%)
Median Age (IQR)	75 (61-78)	73 (68- 77)	74 (66-78)
Mean BMI (Min-Max)	30 (22-49)	29 (18-42)	29 (18-49)
Smoker	1 (7%)	3 (19%)	4 (13%)
Steroid/ immunosuppression use	0 (0%)	0 (0%)	0 (0%)
Diabetes	5 (36%)	4 (25%)	9 (30%)
Connective tissue disorder	1 (7%)	0 (0%)	1 (3%)
COPD	1 (7%)	2 (13%)	3 (10%)
AAA (known or previous repair)	0 (0%)	0 (0%)	0 (0%)
Previous abdominal surgery	8 (57%)	8 (50%)	15 (50%)
Neoadjuvant chemotherapy	1 (7%)	0 (0%)	1 (3%)
Neoadjuvant radiotherapy	1 (7%)	1 (6%)	2 (7%)
Incisional Hernia present pre-operatively	0 (0%)	1 (6%)	1 (3%)
Previous incisional hernia repair	1 (7%)	0 (0%)	1 (3%)
Non incisional hernia present pre-operatively	0 (0%)	3 (19%)	3 (10%)
Mode of surgery			
Laparoscopic	4 (27%)	6 (38%)	10 (33%)
Laparoscopic converted	7 (50%)	3 (19%)	10 (33%)
Open	3 (21%)	7 (44%)	10 (33%)

In arm A, one patient had died prior to 12-month follow-up and a further patient had transferred care to another unit. In arm B, one patient had died prior to 12-month follow-up.

Safety data

There were a total of 16 serious adverse events reported in 10 patients (**Table 2**);

For peer review only

Table 2. Reported serious adverse events

	Arm A	Arm B
Myocardial infarction	2	2
Lower respiratory tract infection	2	1
Pulmonary embolism	1	0
Renal failure	0	1
Anastomotic leak	2	0
Parastomal hernia	0	1
Superficial surgical site infection	2	0
Dehiscence	0	1
Death*	1	0
**Total SAEs**	**10**	**6**
**Total Patients affected**	**5**	**5**

* 1 SAE reported was reported as 'death', therefore it has had to be listed as an event of death. There were two other SAEs that resulted in death within the feasibility study

serious adverse event rate was 34% in arm A and 31% in arm B (p=1.0000). There were no suspected unexpected serious adverse reactions reported in either arm. With regard to wound related complications, there was 1 superficial surgical site infection and 2 organ space surgical site infections reported in arm A and 2 superficial surgical site infections and 1 complete wound dehiscence requiring a return to theatre in arm B (**Table 3**).

For peer review only

Table 3. Wound related complications

	**Arm A**	**Arm B**
Superficial SSI	1	2
Deep SSI	0	0
Organ space SSI	2	0
Wound dehiscence	0	1
**Total wound related complications**	**3**	**3**

The Data Monitoring Committee (DMC) reviewed the un-blinded adverse events data and identified no safety concerns.

For peer review only

Discussion

The results of this feasibility trial demonstrated that a randomised controlled trial designed to compare two suture techniques for the prevention of midline incisional hernia, in patients undergoing cancer resectional surgery, was able to recruit 30 patients over 5 months, as planned. This suggests that the proposed sample size of 800 patients for the main full trial is achievable in the time scale with the proposed number of sites recruiting (approximately 20) [14].

The feasibility trial results established that the trial was acceptable to patients. Patient participation rates were high, demonstrated by 69% of all eligible patients consenting to participation in the trial. Nine patients were screened for eligibility but not consented due to staff shortages highlighting the importance of having adequate number of approved consenting staff on the delegation log. Due to the nature of the study it was accepted that not all patients consented would eventually be randomised. In fact, there was a higher than expected number of patients consented and not eventually randomised (31%). Patients were consented but not randomised if the intraoperative procedure performed did not meet the inclusion criteria, for example not midline incision, conversion to open procedure using a non-midline incision, or emergency patients found not to have a tumour intra-operatively. We acknowledge that this method may increase the risk of selection bias into the trial, however to overcome this we have collected information on the reasons why patients were not randomised after consenting (**Figure 3**).

The setting of the feasibility trial was chosen as the trial's lead site; a high volume teaching hospital. This poses a potential limitation for the main trial at lower volume centres, in terms of ability to recruit participants over the time-period (3 years). However, the sample size required for the pilot and main trial is 800 across roughly 20 sites, which equates to 40 participants required per site and should be achievable over the trial time-period, even for lower volume centres, particularly given the incidence of colorectal cancer within the UK.

The HART trial is a pragmatic trial and as such we are allowing the control (mass closure) arm to be the responsible consultant surgeon's standard closure technique. We acknowledge that this may introduce a degree of variability in the control arm, but in order to counter this our sample size for the main trial has been powered to be able to stratify for site and surgeon.

For peer review only

The serious adverse event rate and wound related complications were similar between both arms and reassuringly there were no suspected unexpected serious adverse reactions reported. It is anticipated that reporting on the full trial will take place in 2019.

For peer review only

Figure 1. Diagram showing the Hughes closure method, using a combination of standard mass closure with a series of horizontal and two vertical mattress sutures within a single suture. When the sutures are pulled to close the defect, the sutures lie both across and along the incision.

Figure 2. HART Study Design

Figure 3. CONSORT diagram

List of abbreviations

AAA: Abdominal aortic aneurysm

BMI: Body Mass Index

CONTINT: CONTinuous versus INTerrupted abdominal wall closure after emergency midline laparotomy

COPD: Chronic Obstructive Pulmonary Disease

CT: Computed tomography

DMC: Data Monitoring Committee

HART: Hughes Abdominal Repair Trial

IQR: interquartile range

NIHR: National Institute of Health Research

PDS: polydioxanone

SAEs: Serious Adverse Events

SSI: Surgical Site Infection

STITCH: Suture Techniques to reduce the Incidence of The inCisional Hernia (RCT)

For peer review only

Competing Interests

There are no competing interests to declare

Authors Contributions

RLH, JC, DB, BR, ITR, JT conceived, designed and drafted the initial protocol

JH aided in redrafting and revising the protocol and contributed heavily to completion of the feasibility trial and pilot stages

SI, AW, NB, ITR have written and designed the analysis plan and revised the protocol

JC, DB, ITR and JT led the team that acquired the funding

RLH wrote the first draft of the manuscript

All authors agree to be accountable for all aspects of the work. All authors read and approved the final manuscript.

References

1. Bosanquet D, Ansell J, Abdelrahman T, Cornish J, Harries R, Stimpson A, Davies L, Glasbey J, Frewer K, Frewer N, Russell D, Russell I, Torkington J. Systematic Review and Meta-Regression of factors affecting midline incisional hernia rates: Analysis of 14,618 patients. PLoS ONE 2015;10(9): e0138745.

2. Pereira JA, Pera M, Grande L. [Incidence of incisional hernia after open and laparoscopic colorectal cancer resection]. Cir Esp. 2013;91(1):44-9.

3. van Ramshorst GH, Eker HH, Hop WC, Jeekel J, Lange JF. Impact of incisional hernia on health-related quality of life and body image: a prospective cohort study. Am J Surg, 2012;204(2):144-50.

4. Diener MK, Voss S, Jensen K, Büchler MW, Seiler CM. Elective midline laparotomy closure: the INLINE systematic review and meta-analysis. Ann Surg, 2010; 251(5):843-56.

5. van 't Riet M, Steyerberg EW, Nellensteyn J, Bonjer HJ, Jeekel J. Meta-analysis of techniques for closure of midline abdominal incisions. Br J Surg, 2002;89(11):1350-6.

6. Sajid MS, Parampalli U, Baig MK, McFall MR. A systematic review on the effectiveness of slowly-absorbable versus non-absorbable sutures for abdominal fascial closure following laparotomy. Int J Surg, 2011; 9(8):615-25.

For peer review only

7. Deerenberg EB, Harlaar JJ, Steyerberg EW, Lont HE, van Doorn HC, Heisterkamp J, Wijnhoven BP, Schouten WR, Cense HA, Stockmann HB, Berends FJ, Dijkhuizen FP, Dwarkasing RS, Jairam AP, van Ramshorst GH, Kleinrensink GJ, Jeekel J, Lange JF. Small bites versus large bites for closure of abdominal midline incisions (STITCH): a double-blind, multicentre, randomised controlled trial. Lancet. 2015, 386(10000):1254-60.

8. Rahbari NN, Knebel P, Kieser M, Bruckner T, Bartsch DK, Friess H, Mihaljevic AL, Stern J, Diener MK, Voss S, Rossion I, Büchler MW, Seiler CM. Design and current status of CONTINT: continuous versus interrupted abdominal wall closure after emergency midline laparotomy - a randomized controlled multicenter trial [NCT00544583]. Trials, 2012;13:72.

9. Muysoms FE, Antoniou SA, Bury K, Campanelli G, Conze J, Cuccurullo D, Beaux AC, Deerenberg EB, East B, Fortelny RH, Gillion J, Henriksen NA, Israelsson L, Jairam A, Jänes A, Jeekel J, López-Cano M, Miserez M, Morales-Conde S, Sanders DL, Simons MP, Śmietański M, Venclauskas L, Berrevoet F; EuropeanHernia Society. European Hernia Society guidelines on the closure of abdominal wall incisions. Hernia 2015;19:1–24.

10. Hughes BR & Webster D. Leslie Ernest Hughes OBITUARY. BMJ, 2011. 343.

11. Shukla VK, Gupta A, Singh H, Pandey M, Gautam A. Cardiff repair of incisional hernia: a university hospital experience. Eur J Surg, 1998;164(4):271-4.

12. Mudge M, Harding KG, Hughes LE. Incisional hernia. Br J Surg, 1986;73(1): 82.

13. Godara R, Garg P, Shankar G. Comparative evaluation of Cardiff repair and mesh plasty in incisional hernias. Internet J Surg [Internet]. 2007; 9 [about 8 p.].

14. Malik R & Scott NA. Double near and far prolene suture closure: a technique for abdominal wall closure after laparostomy. Br J Surg, 2001;88(1):146-7.

15. Cornish J, Harries RL, Bosanquet D, Rees B, Ansell J, Frewer N, Dhruva Rao P, Parry C, Ellis-Owen R, Phillips SM, Morris C, Horwood J, Davies ML, Davies MM, Hargest R, Hilton J, Harris D, Ben-Sassi A, Rajagopal R, Hanratty D, Islam S, Watkins A, Bashir N, Jones S, Russell IR, Torkington J, on behalf of the HART Trial Management Group. Hughes Abdominal Repair Trial (HART) - Abdominal wall closure techniques to reduce the incidence of incisional hernias:

For peer review only

Study protocol for a multi-centre pragmatic randomised controlled trial (ISRCTN 25616490). Trials 2016; 17(1):454.

16. Russell D, Hoare ZSJ, Whitaker Rh, Whitaker CJ, Russell IT. Generalised method for adaptive randomisation in clinical trials. Statistics in Medicine 2011; 30: 922-34.

For peer review only

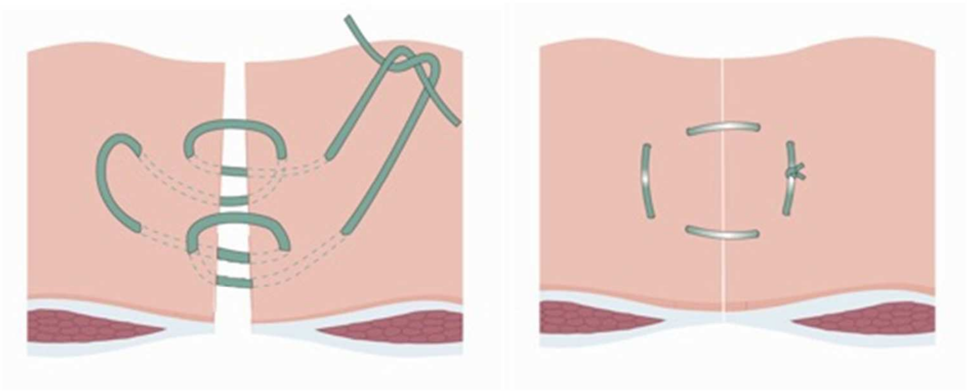

Figure 1. Diagram showing the Hughes closure method, using a combination of standard mass closure with a series of horizontal and two vertical mattress sutures within a single suture. When the sutures are pulled to close the defect, the sutures lie both across and along the incision.

45x19mm (300 x 300 DPI)

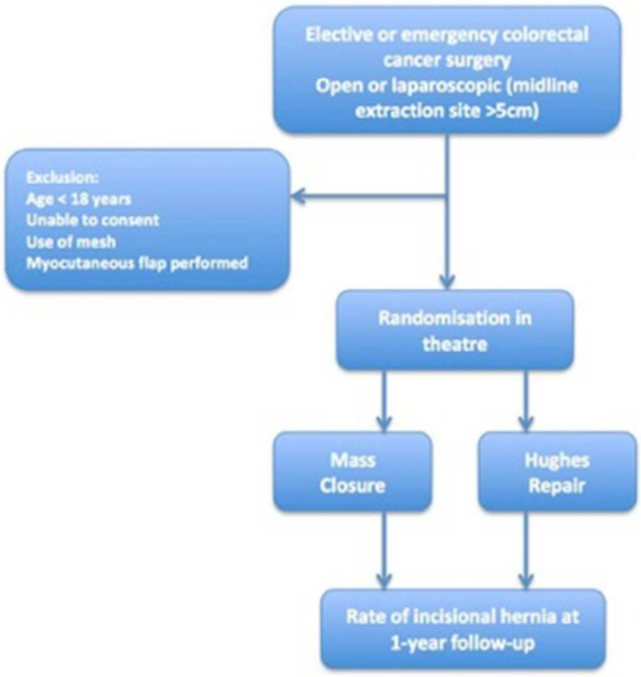

Figure 2. HART Study Design

43x31mm (300 x 300 DPI)

For peer review only

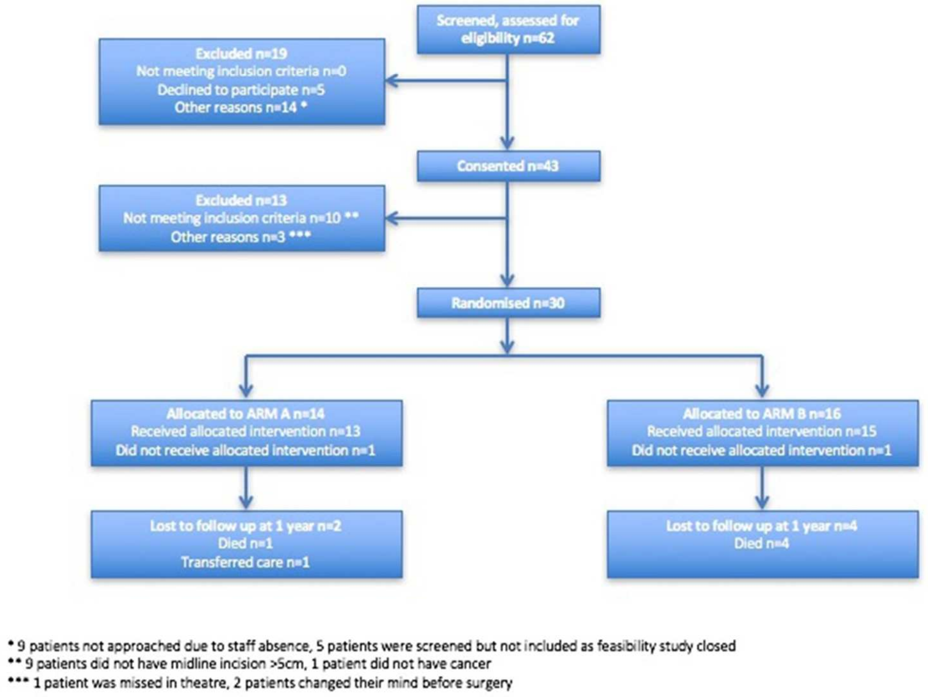

Figure 3. CONSORT diagram

53x39mm (300 x 300 DPI)

review only

CONSORT 2010 checklist of information to include when reporting a randomised trial*

Section/Topic	Item No	Checklist item	Reported on page No
**Title and abstract**			
	1a	Identification as a randomised trial in the title	1
	1b	Structured summary of trial design, methods, results, and conclusions (for specific guidance see CONSORT for abstracts)	2
**Introduction**			
Background and objectives	2a	Scientific background and explanation of rationale	4-5
	2b	Specific objectives or hypotheses	4-6
**Methods**			
Trial design	3a	Description of trial design (such as parallel, factorial) including allocation ratio	6
	3b	Important changes to methods after trial commencement (such as eligibility criteria), with reasons	n/a
Participants	4a	Eligibility criteria for participants	6-7
	4b	Settings and locations where the data were collected	6-7
Interventions	5	The interventions for each group with sufficient details to allow replication, including how and when they were actually administered	7-8
Outcomes	6a	Completely defined pre-specified primary and secondary outcome measures, including how and when they were assessed	n/a
	6b	Any changes to trial outcomes after the trial commenced, with reasons	n/a
Sample size	7a	How sample size was determined	8
	7b	When applicable, explanation of any interim analyses and stopping guidelines	n/a
Randomisation:			
Sequence generation	8a	Method used to generate the random allocation sequence	7
	8b	Type of randomisation; details of any restriction (such as blocking and block size)	7
Allocation concealment mechanism	9	Mechanism used to implement the random allocation sequence (such as sequentially numbered containers), describing any steps taken to conceal the sequence until interventions were assigned	7
Implementation	10	Who generated the random allocation sequence, who enrolled participants, and who assigned participants to interventions	7
Blinding	11a	If done, who was blinded after assignment to interventions (for example, participants, care providers, those	7

For peer review only - http://bmjopen.bmj.com/site/about/guidelines.xhtml

		assessing outcomes) and how	
	11b	If relevant, description of the similarity of interventions	
Statistical methods	12a	Statistical methods used to compare groups for primary and secondary outcomes	n/a
	12b	Methods for additional analyses, such as subgroup analyses and adjusted analyses	n/a
**Results**			
Participant flow (a diagram is strongly recommended)	13a	For each group, the numbers of participants who were randomly assigned, received intended treatment, and were analysed for the primary outcome	9
	13b	For each group, losses and exclusions after randomisation, together with reasons	9
Recruitment	14a	Dates defining the periods of recruitment and follow-up	9
	14b	Why the trial ended or was stopped	9
Baseline data	15	A table showing baseline demographic and clinical characteristics for each group	14
Numbers analysed	16	For each group, number of participants (denominator) included in each analysis and whether the analysis was by original assigned groups	9
Outcomes and estimation	17a	For each primary and secondary outcome, results for each group, and the estimated effect size and its precision (such as 95% confidence interval)	n/a
	17b	For binary outcomes, presentation of both absolute and relative effect sizes is recommended	n/a
Ancillary analyses	18	Results of any other analyses performed, including subgroup analyses and adjusted analyses, distinguishing pre-specified from exploratory	n/a
Harms	19	All important harms or unintended effects in each group (for specific guidance see CONSORT for harms)	n/a
**Discussion**			
Limitations	20	Trial limitations, addressing sources of potential bias, imprecision, and, if relevant, multiplicity of analyses	10
Generalisability	21	Generalisability (external validity, applicability) of the trial findings	10
Interpretation	22	Interpretation consistent with results, balancing benefits and harms, and considering other relevant evidence	10
**Other information**			
Registration	23	Registration number and name of trial registry	1
Protocol	24	Where the full trial protocol can be accessed, if available	6
Funding	25	Sources of funding and other support (such as supply of drugs), role of funders	8

*We strongly recommend reading this statement in conjunction with the CONSORT 2010 Explanation and Elaboration for important clarifications on all the items. If relevant, we also recommend reading CONSORT extensions for cluster randomised trials, non-inferiority and equivalence trials, non-pharmacological treatments, herbal interventions, and pragmatic trials. Additional extensions are forthcoming: for those and for up to date references relevant to this checklist, see www.consort-statement.org.

For peer review only

For peer review only - http://bmjopen.bmj.com/site/about/guidelines.xhtml
